# circ_0000132 Regulates Chicken Granulosa Cell Proliferation Apoptosis and E2/P4 Synthesis via miR-206 E2F5 Signaling

**DOI:** 10.3390/ijms262110779

**Published:** 2025-11-05

**Authors:** Huanqi Yang, Wei Li, Guanhua Fu, Sihan Liu, Tenghe Ma

**Affiliations:** 1 College of Life Sciences and Food Engineering, Hebei University of Engineering, Handan 056038, China; yanghq947@163.com (H.Y.); guanhua1220@163.com (G.F.); sihanliu1234@163.com (S.L.); 2 College of Clinical Medicine, Hebei University of Engineering, Handan 056038, China; 15945196231@163.com

**Keywords:** follicle selection, circFBN1, granulosa cells, proliferation, apoptosis, steroid hormones

## Abstract

This study investigates the regulatory role of circFBN1 in chicken follicular granulosa cells (GCs) and its underlying molecular mechanisms through the miR-206/E2F5 pathway. circFBN1 was found to significantly enhance GC proliferation and inhibit apoptosis, as evidenced by increased expression of proliferation-related genes (PCNA, CDK1, and CCND1) and decreased expression of apoptosis-related genes (Caspase-3). Additionally, circFBN1 overexpression promoted the secretion of estradiol (E2) and progesterone (P4) by upregulating steroidogenesis-related genes (StAR and CYP11A1). Mechanistic studies revealed that circFBN1 functions as a molecular sponge for miR-206, thereby alleviating its inhibitory effect on the target gene E2F5. Dual-luciferase reporter assays confirmed the specific binding between circFBN1 and miR-206. Overexpression of miR-206 had the opposite effects, inhibiting GC proliferation, inducing apoptosis, and reducing E2 and P4 secretion by downregulating StAR and CYP11A1. Furthermore, E2F5 was identified as a direct target of miR-206, and its knockdown significantly reduced GC proliferation, increased apoptosis, and decreased steroid hormone secretion. These findings elucidate the regulatory mechanisms of the circFBN1/miR-206/E2F5 axis in avian follicle development and provide potential molecular targets for improving poultry reproductive performance. Future research should focus on exploring the upstream regulators of this axis and its interactions with other signaling pathways.

## 1. Introduction

The development and selection of ovarian follicles in poultry constitute critical determinants of egg production performance, with their regulatory mechanisms representing a central focus of reproductive biology research [1]. Traditional investigations have primarily centered on classical endocrine pathways mediated by gonadotropins (follicle-stimulating hormone [FSH] and luteinizing hormone [LH]) and their receptors [2,3,4]; however, recent discoveries concerning non-coding RNAs—particularly circular RNAs (circRNAs) and microRNAs (miRNAs)—and their competitive endogenous RNA (ceRNA) regulatory networks have provided novel perspectives for elucidating the molecular mechanisms governing follicular development [5,6,7,8]. circRNAs, characterized by their covalently closed circular structure formed through back-splicing, exhibit remarkable stability and tissue-specific expression patterns, positioning them as pivotal regulators of gene expression [9,10]. In contrast to circRNAs, miRNAs are small single-stranded ncRNAs that exert post-transcriptional control by binding to the 3′ untranslated regions (3’UTRs) of target mRNAs [11,12,13], leading to mRNA degradation or translational repression [14,15,16]. In the context of follicular development, miRNAs modulate essential cellular processes, including GC proliferation, apoptosis, and steroidogenesis. While circRNA/miRNA/mRNA regulatory networks have been demonstrated in mammals to regulate key reproductive processes such as germ cell development, follicular atresia, and steroid hormone metabolism, analogous investigations in avian species—particularly commercial laying hens—remain comparatively nascent.

Folliculogenesis represents a dynamic continuum initiated by primordial follicle activation, progressing through growth and maturation phases, and culminating in ovulation [17,18]. This process is orchestrated by the hypothalamic–pituitary–ovarian (HPO) axis, wherein FSH and LH serve as principal hormonal regulators [19,20]. These gonadotropins activate corresponding receptors on granulosa cells, triggering signaling cascades that promote cellular proliferation, estrogen synthesis, and follicular fluid accumulation [21,22,23]. As follicles advance in development, granulosa cells differentiate to acquire steroidogenic capacity, with estrogen production subsequently modulating gonadotropin secretion via feedback mechanisms to maintain hormonal homeostasis [24,25]. The follicular selection phase represents a critical juncture wherein a subset of developing follicles is recruited into a rapid growth trajectory while others undergo atresia [26]. This selection process involves intricate paracrine and autocrine regulatory networks, with insulin-like [27,28,29] growth factors (IGFs) and transforming growth factors (TGFs) [30,31] acting synergistically with endocrine hormones to coordinate follicular fate determination [32,33,34]. Granulosa cells, as the functional cornerstone of follicles, regulate follicular destiny through their proliferative/apoptotic equilibrium and steroidogenic competence, thereby serving as pivotal regulatory hubs in follicular selection.

The regulatory roles of non-coding RNAs in folliculogenesis have garnered increasing attention. circRNAs function as molecular sponges for miRNAs, competitively binding these small RNAs to derepress target gene expression [35,36,37]. miRNAs, in turn, modulate follicular development by targeting key genes involved in steroidogenesis and cell cycle regulation [38,39,40]. While mammalian studies have established the involvement of circRNA/miRNA/mRNA networks in granulosa cell function, follicular atresia, and hormone synthesis, comparable investigations in avian models remain limited. Given the centrality of GCs to follicular development and the emerging role of circRNAs in reproductive regulation, we previously conducted high-throughput circRNA sequencing on chicken SYFs and LYFs—key stages of follicular selection—to identify differentially expressed circRNAs with potential regulatory functions. Among these candidates, circ_0000132 (As it originates from exons 3–6 of the fibrin 1 [FBN1] gene, it is collectively referred to as circFBN1 in the following text.) was found to be significantly upregulated in LYFs compared to SYFs. FBN1 itself encodes a structural glycoprotein that polymerizes into microfibers, which contribute to cell proliferation and tissue integrity [41,42]; however, the function of its circular derivative, circFBN1, in avian folliculogenesis has not been explored. The present study employs the laying hen as an experimental model, utilizing high-throughput sequencing to identify follicle development-associated circRNAs, with particular emphasis on elucidating the functional role of the circFBN1/miR-206/E2F5 regulatory axis in granulosa cell proliferation, apoptosis, and steroid hormone synthesis. Our findings reveal that circFBN1 exhibits differential expression during follicular selection and may regulate E2F5 expression by sequestering miR-206, thereby influencing granulosa cell function. miR-206, a key miRNA implicated in cellular proliferation and apoptosis, and E2F5, a transcription factor involved in cell cycle regulation and hormone synthesis, collectively form a regulatory axis whose discovery not only expands the theoretical framework of non-coding RNA-mediated reproductive regulation in avian species but also identifies potential molecular targets for genetic improvement of poultry reproductive performance.

The significance of this research lies in its elucidation of circRNA/miRNA/mRNA regulatory mechanisms in avian folliculogenesis, offering novel insights into non-coding RNA networks within the avian reproductive system. Theoretically, this work extends the ceRNA paradigm to avian follicular development, thereby enriching the functional repertoire of circRNAs in reproductive biology. Practically, modulation of circFBN1 expression may represent a viable strategy for optimizing ovulation cycles and reducing follicular atresia in laying hens, thereby enhancing reproductive efficiency. Future investigations should focus on delineating the upstream and downstream components of the circFBN1/miR-206/E2F5 axis and characterizing its spatiotemporal expression dynamics across different follicular stages. Such endeavors will facilitate the development of molecular tools for improving egg production performance in poultry breeding programs.

## 2. Results

### 2.1. circ_0000132 Affects GC Proliferation, Apoptosis, and Steroid Synthesis and Secretion

**Figure 1:**
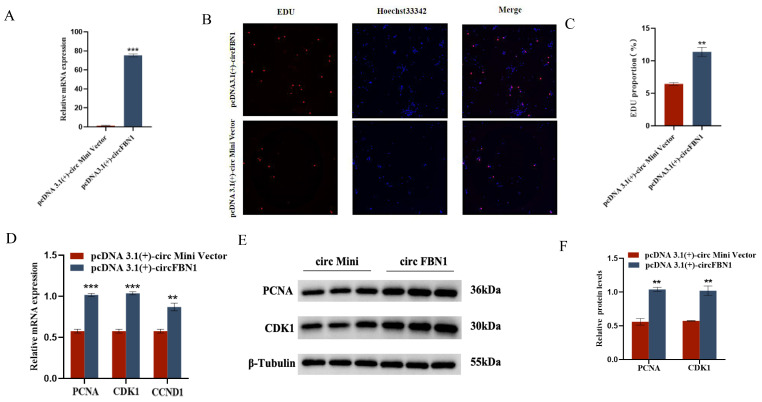
circFBN1 affects GC proliferation, apoptosis, and steroid synthesis and secretion. (**A**) Overexpression efficiency of circFBN1 in GCs. (**B**,**C**) EdU staining and positive EdU cell rate in GCs after circFBN1 overexpression. (**D**) Relative mRNA expression levels of key genes related to cell proliferation (PCNA, CDK1, and CCND1) in GCs after circFBN1 overexpression. (**E**,**F**) Relative protein expression levels of key genes related to cell proliferation (PCNA and CDK1) in GCs after circFBN1 overexpression. ** *p* < 0.01; *** *p* < 0.001.

**Figure 2:**
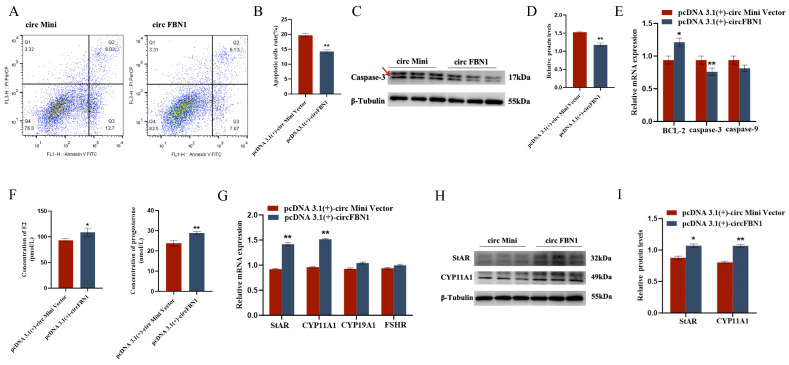
circFBN1 affects GC proliferation, apoptosis, and steroid synthesis and secretion. (**A**,**B**) Flow cytometry results of GCs after circFBN1 overexpression. (**C**,**D**) Relative protein expression levels of key genes related to cell apoptosis (Caspase-3) in GCs after circFBN1 overexpression (The areas marked with arrows are the target strips.). (**E**) The relative protein expression levels of key genes related to apoptosis in GCs after overexpression of circFBN1. (**F**) Concentrations of E2 and P4 in GCs after circFBN1 overexpression. (**G**) Relative mRNA expression levels of key genes related to steroid synthesis in GCs after circFBN1 overexpression. (**H**,**I**) Relative protein expression levels of key genes related to steroid synthesis (StAR and CYP11A1) in GCs after circFBN1 overexpression. * *p* < 0.05; ** *p* < 0.01.

We first detected the overexpression of the circFBN1 plasmid transfection, and the detection results were successful in overexpression, which allows us to proceed with subsequent experiments. The EDU cell proliferation assay and flow cytometry results showed that circFBN1 could significantly promote the proliferation of chicken follicular granulosa cells and inhibit their apoptosis. Specifically, after overexpression of circFBN1, the expression levels of proliferation-related genes PCNA, CDK1, and CCND1 in granulosa cells were significantly upregulated, while the expression level of apoptosis-related gene Caspase-3 was significantly downregulated, and the expression level of anti-apoptotic gene BCL-2 was significantly upregulated. In addition, circFBN1 also significantly promoted the secretion of estradiol and progesterone in granulosa cells. After overexpression, the expression levels of steroidogenesis-related genes StAR and CYP11A1 were significantly upregulated, but there were no significant changes in the expression of CYP19A1 and FSHR (Figure 1 and Figure 2).

### 2.2. circ_0000132 Acted as a Sponge of gga-miR-206

**Figure 3:**
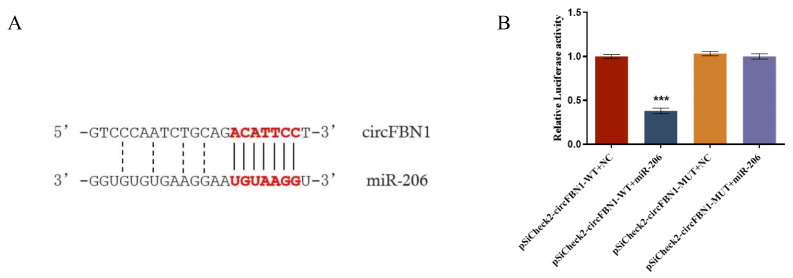
circFBN1 acted as a sponge of gga-miR-206: (**A**) Prediction of binding sites of circFBN1 and gga-miR-206; (**B**) Double luciferase report of gga-miR-206. *** *p* < 0.001.

Bioinformatics prediction suggested that gga-miR-206 may be a target miRNA of circFBN1. To further investigate the effects of gga-miR-206 on the potential ceRNA network of circFBN1, we constructed wild-type and mutant vectors of circFBN1 and subsequently determined the expression of gga-miR-206 in GCs. The results indicated that circFBN1 acts as a molecular sponge for miR-206 through a competitive endogenous RNA (ceRNA) mechanism, thereby relieving the inhibitory effect of miR-206 on its target genes. The dual-luciferase reporter assay verified the specific binding mechanism between circFBN1 and miR-206 (Figure 3).

### 2.3. gga-miR-206 Affects GC Proliferation, Apoptosis, and Steroid Synthesis and Secretion

**Figure 4:**
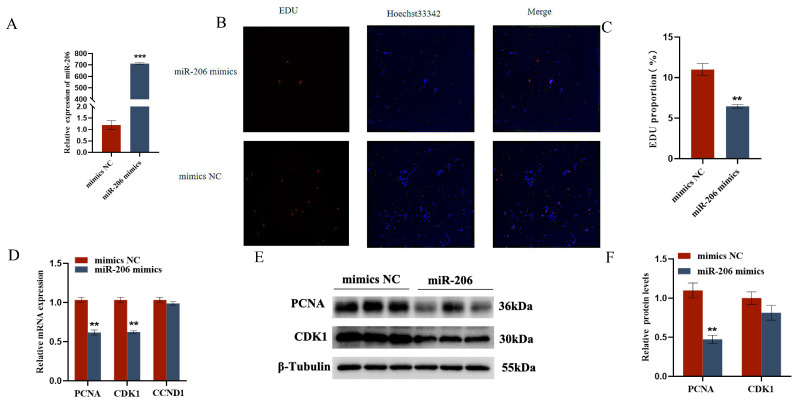
gga-miR-206 affects GC proliferation, apoptosis, and steroid synthesis and secretion: (**A**) Detection of transfection efficiency of miR-206 mimics; (**B**,**C**) Effect of gga-miR-206 on the proliferation of chicken GCs; (**D**) Expression levels of genes related to cell proliferation (CDK1, PCNA, CCND1); (**E**,**F**) Protein expression changes of CDK1 and PCNA after overexpression of gga-miR-206. ** *p* < 0.01; *** *p* < 0.001.

**Figure 5:**
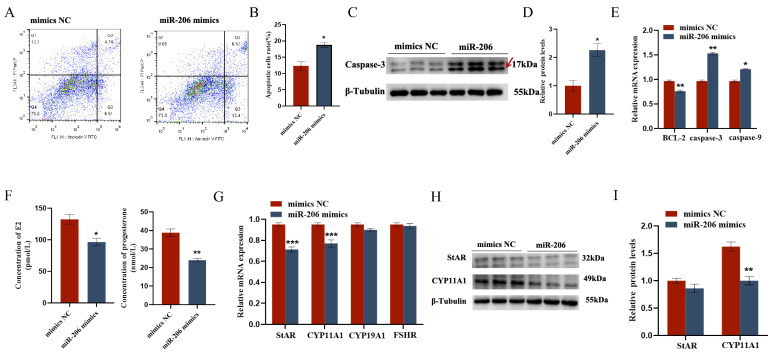
gga-miR-206 affects GC proliferation, apoptosis, and steroid synthesis and secretion: (**A**,**B**) Effect of gga-miR-206 on apoptosis of chicken GCs; (**C**,**D**) Protein expression changes of Caspase-3 after treatment with overexpression of gga-miR-206 (The areas marked with arrows are the target strips.); (**E**) Expression levels of genes related to cell proliferation (BCL-2, Caspase-3, Caspase-9); (**F**) ELISA was used to detect the effect of gga-miR-206 on the synthesis of estradiol and progesterone in chicken GCs; (**G**) Expression levels of genes related to steroid synthesis (StAR, CYP11A1, CYP19A1, FSHR); (**H**,**I**) Protein expression changes of StAR and CYP11A1 after overexpression of gga-miR-206. * *p* < 0.05; ** *p* < 0.01; *** *p* < 0.001.

The EDU cell proliferation assay and flow cytometry results showed that gga-miR-206 significantly inhibited the proliferation of chicken follicular granulosa cells and promoted their apoptosis. After overexpression of gga-miR-206, the expression levels of proliferation-related genes PCNA and CDK1 in granulosa cells were significantly downregulated, while the expression level of apoptosis-related gene Caspase-3 was significantly upregulated, and the expression level of anti-apoptotic gene BCL-2 was significantly downregulated. In addition, gga-miR-206 also significantly inhibited the secretion of estradiol and progesterone in granulosa cells. After overexpression, the expression levels of steroidogenesis-related genes StAR and CYP11A1 were significantly downregulated, but there were no significant changes in the expression of CYP19A1 and FSHR (Figure 4 and Figure 5).

### 2.4. gga-miR-206 Targets the E2F5 Gene

**Figure 6:**
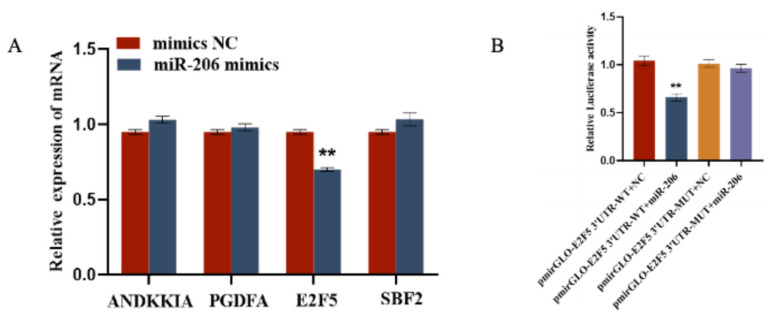
gga-miR-206 targets the E2F5 Gene: (**A**) Verification of target relationship between miR-206 and E2F5; (**B**) Validation of the targeting relationship between miR-206-3p and E2F5 3′UTR. ** *p* < 0.01.

Using the TargetScan (Release 8.0) online software, we screened for mRNAs related to the function of circFBN1 and ultimately identified four target genes of miR-206, namely ANKKIA, PDGFA, E2F5, and SBF2. After transfecting gga-miR-206 mimics, we measured the expression of these four genes in GCs. The results showed that gga-miR-206 inhibited the expression of E2F5, while having no effect on the expression of ANKKIA, PDGFA, and SBF2. Additionally, we used a dual-luciferase reporter assay to test the target relationship with gga-miR-206. The results showed that, compared with other treatments, the relative luciferase activity of E2F5 WT treated with gga-miR-206 mimics was significantly reduced, indicating that gga-miR-206 can directly bind to E2F5 (Figure 6).

### 2.5. E2F5 Affects GC Proliferation, Apoptosis, and Steroid Synthesis and Secretion

**Figure 7:**
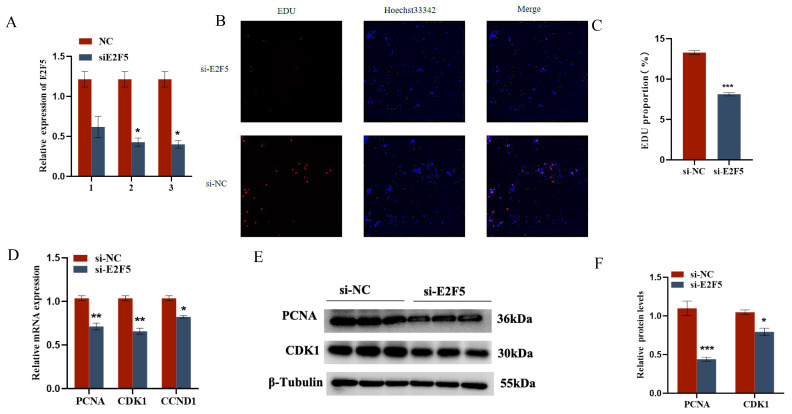
E2F5 affects GC proliferation, apoptosis, and steroid synthesis and secretion: (**A**) The interference efficiency of E2F5; (**B**,**C**) Validation of the targeting relationship between miR-206-3p and E2F5 3′UTR. Effect of E2F5 on the proliferation of GCs; (**D**) Expression levels of genes related to cell proliferation (CDK1, PCNA, CCND1); (**E**,**F**) Protein expression changes of CDK1 and PCNA after interference E2F5 treatment. * *p* < 0.05; ** *p* < 0.01; *** *p* < 0.001.

**Figure 8:**
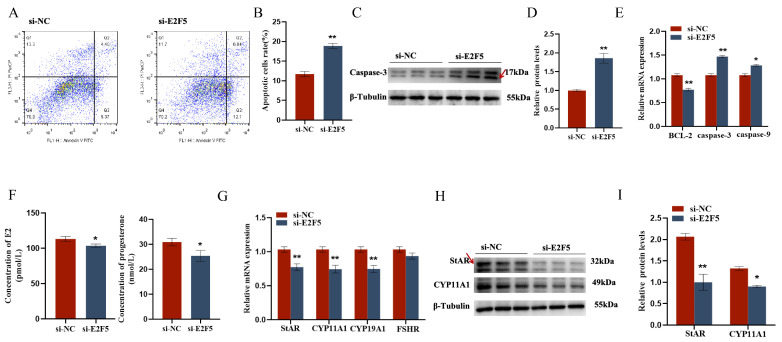
E2F5 affects GC proliferation, apoptosis, and steroid synthesis and secretion: (**A**,**B**) Effect of gga-miR-206 on apoptosis of GCs; (**C**,**D**) Change of Caspase-3 protein expression after interference E2F5 treatment (The areas marked with arrows are the target strips); (**E**) Expression levels of genes related to cell proliferation (BCL-2, Caspase-3, Caspase-9); (**F**) The effect of si-E2F5 on estradiol and progesterone synthesis in chicken GCs was determined by ELISA; (**G**) Expression levels of genes related to steroid synthesis (StAR, CYP11A1, CYP19A1, FSHR); (**H**,**I**) The protein expression of StAR, CYP11A1 changes after interfering with E2F5 (The areas marked with arrows are the target strips.). * *p* < 0.05; ** *p* < 0.01.

The target gene E2F5 exerts significant regulatory functions in chicken follicular granulosa cells. On one hand, it markedly promotes the proliferation of granulosa cells and inhibits their apoptosis. This is evidenced by the significant downregulation of proliferation-related genes PCNA, CDK1, and CCND1, and the significant upregulation of apoptosis-related genes Caspase-3 and Caspase-9 following E2F5 knockdown, along with a significant decrease in the expression level of the anti-apoptotic gene BCL-2. On the other hand, E2F5 also significantly enhances the secretion of estradiol and progesterone in granulosa cells. Upon E2F5 knockdown, the expression levels of steroidogenesis-related genes StAR, CYP11A1, and CYP19A1 are significantly reduced, leading to a marked decrease in the secretion of estradiol and progesterone (Figure 7 and Figure 8).

## 3. Discussion

The ovary is the core organ regulating hen egg-laying performance, and follicular development—especially the precise control of follicular selection, growth, maturation, and atresia—directly determines the quantity and quality of eggs produced. Steroid hormones, particularly estradiol (E2) and progesterone (P4), are key signaling molecules throughout the ovulation process: they not only drive follicular growth and maturation but also maintain the rhythmicity of the egg-laying cycle by modulating the hypothalamic–pituitary–ovarian (HPO) axis [43]. Granulosa cells (GCs), as the primary functional cells of follicles, are responsible for steroid hormone synthesis—they regulate cholesterol transport and the expression of steroidogenic enzymes to control E2 and P4 secretion, making them the “functional core” of follicular development. In recent years, circular RNAs (circRNAs), once considered “transcriptional noise,” have been identified as crucial regulators of gene expression in reproductive biology due to their stable circular structure and tissue-specific expression patterns [44,45], and this study systematically explored the function and mechanism of circFBN1 in chicken follicular GCs, with a focus on interpreting how its regulatory effects on GC proliferation, apoptosis, and steroid synthesis support follicular development, thereby bridging the gap between non-coding RNA function and avian reproductive performance.

Our first key finding was that circFBN1 significantly promotes chicken GC proliferation and inhibits apoptosis, while also enhancing E2 and P4 secretion, which directly links circFBN1 to two core processes of follicular development: follicular survival (via balancing GC proliferation and apoptosis) and follicular maturation (via supporting steroid synthesis). From the perspective of follicular selection—a critical juncture where only a subset of small yellow follicles (SYFs) is recruited to become large yellow follicles (LYFs) while others undergo atresia—the functional role of circFBN1 becomes particularly meaningful. EdU proliferation assays and flow cytometry showed that overexpressing circFBN1 increased the proportion of proliferating GCs and reduced apoptotic rates, which was further validated by molecular markers: proliferation-related genes (PCNA, CDK1, CCND1) were upregulated, the apoptosis-related gene Caspase-3 was downregulated, and the anti-apoptotic gene BCL-2 was upregulated. PCNA is a marker of cell cycle progression, CDK1 regulates the G2/M phase transition, and CCND1 controls the G1/S phase transition—their upregulation indicates that circFBN1 accelerates the GC cell cycle to provide sufficient cellular mass for follicular growth, while the downregulation of Caspase-3 (a key executor of apoptosis) and upregulation of BCL-2 (an inhibitor of mitochondrial apoptotic pathways) suggest that circFBN1 suppresses GC apoptosis to prevent premature follicular atresia. In terms of steroid synthesis, ELISA results showed that circFBN1 overexpression significantly increased E2 and P4 secretion, accompanied by upregulated expression of steroidogenic genes StAR and CYP11A1 (no significant changes were observed in CYP19A1 and FSHR). This specificity in gene regulation provides important clues about circFBN1’s mechanism of action: StAR is responsible for transporting cholesterol (the precursor of steroid hormones) into the mitochondria—the rate-limiting step of steroid synthesis—while CYP11A1 catalyzes the conversion of cholesterol to pregnenolone (the first committed step in steroidogenesis). Their upregulation directly explains the increased E2 and P4 production, indicating that circFBN1 enhances GC steroidogenic capacity by targeting the early steps of the steroid synthesis pathway. The lack of effect on CYP19A1 (which converts androgens to estrogens) and FSHR (a receptor for follicle-stimulating hormone) suggests that circFBN1 does not interfere with downstream estrogen synthesis or gonadotropin signaling, but rather acts on the upstream, rate-limiting steps of steroidogenesis, ensuring it can enhance hormone secretion without disrupting the HPO axis’s feedback balance—a key factor for maintaining stable follicular development. Additionally, our GWAS-based analysis found that circFBN1 is differentially expressed between SYFs and LYFs, with higher expression in LYFs. This expression pattern aligns with its functional role: LYFs are follicles that have successfully passed the selection phase and entered rapid growth, requiring high GC proliferation rates and robust steroid synthesis. The higher expression of circFBN1 in LYFs further supports its role as a “promoter” of follicular selection and maturation.

A central question in this study was how circFBN1 exerts its regulatory effects on GCs, and our mechanistic investigations confirmed that circFBN1 acts as a molecular sponge for gga-miR-206, following the competitive endogenous RNA (ceRNA) paradigm. This finding is critical for understanding the specificity of circFBN1’s function, as it identifies miR-206 as the intermediate through which circFBN1 modulates downstream gene expression. Bioinformatics predictions first suggested a potential binding site between circFBN1 and miR-206, and dual-luciferase reporter assays validated this interaction: co-transfecting circFBN1 wild-type vectors with miR-206 mimics significantly reduced luciferase activity, while mutating the binding site abolished this effect, directly confirming specific binding—a prerequisite for the ceRNA mechanism. The ceRNA model proposes that circRNAs sequester miRNAs to reduce their availability for binding target mRNAs, thereby derepressing target gene expression; for circFBN1, this means sponging miR-206 to relieve its inhibitory effect on downstream target genes (subsequently identified as E2F5), indirectly promoting GC proliferation, inhibiting apoptosis, and enhancing steroid synthesis. To further verify this mechanism, we analyzed the functional effects of miR-206 overexpression and found they were the exact opposite of circFBN1 overexpression: miR-206 mimics significantly inhibited GC proliferation (downregulating PCNA and CDK1), induced apoptosis (upregulating Caspase-3 and downregulating BCL-2), and reduced E2 and P4 secretion (downregulating StAR and CYP11A1). This “reverse effect” strongly supports the ceRNA relationship—if circFBN1 acts by sponging miR-206, then increasing miR-206 levels (via mimics) should overwhelm circFBN1’s sponging capacity, restoring miR-206’s inhibitory effects on GC function. The consistency between miR-206’s functional phenotype and the inverse of circFBN1’s phenotype confirms that miR-206 is a downstream mediator of circFBN1, and that their interaction is not arbitrary but functionally relevant to GC biology. Notably, miR-206 has been previously implicated in cell proliferation and apoptosis in mammalian systems (e.g., inhibiting proliferation and promoting apoptosis in cancer cells), and our findings in chicken GCs suggest that miR-206’s role in regulating cell fate is evolutionarily conserved, while also expanding its function to include the regulation of steroid hormone synthesis—highlighting its importance as a key regulator of reproductive cell function across species and making it a potential target for manipulating reproductive performance in both mammals and poultry.

To fully understand the circFBN1/miR-206 pathway, we next identified the downstream mRNA target of miR-206. Using DAVID functional annotation, we screened four potential targets (ANKK1A, PDGFA, E2F5, SBF2) and found that only E2F5 expression was significantly inhibited by miR-206 overexpression—indicating that E2F5 is a specific target of miR-206 in chicken GCs. Dual-luciferase reporter assays further confirmed this: miR-206 mimics reduced luciferase activity of E2F5 wild-type vectors (but not mutant vectors), proving direct binding between miR-206 and the 3’UTR of E2F5. Functional analysis of E2F5 further closed the regulatory loop of the circFBN1/miR-206/E2F5 axis. E2F5 is a transcription factor known to regulate cell cycle progression and gene expression, which is consistent with our findings that interfering with E2F5 (via siRNA) significantly inhibited GC proliferation (downregulating PCNA, CDK1, CCND1) and induced apoptosis (upregulating Caspase-3, Caspase-9; downregulating BCL-2). These effects are identical to those of miR-206 overexpression, confirming that E2F5 is a functional target of miR-206: miR-206 inhibits GC proliferation and promotes apoptosis by suppressing E2F5. Additionally, E2F5 knockdown reduced E2 and P4 secretion and downregulated StAR, CYP11A1, and even CYP19A1 (unlike miR-206 or circFBN1, which had no effect on CYP19A1), suggesting that E2F5 may act as a transcription factor regulating multiple steroidogenic genes—including not only the early steps (StAR, CYP11A1) but also downstream estrogen synthesis (CYP19A1). The broader effect of E2F5 on steroidogenic genes indicates that it is a central regulator of GC steroid synthesis, and that miR-206’s inhibition of E2F5 is the key mechanism by which it reduces hormone secretion. Collectively, these results allow us to propose a complete regulatory model: circFBN1, by sponging miR-206, prevents miR-206 from binding to E2F5’s 3’UTR, thereby maintaining high E2F5 expression. E2F5 then promotes GC proliferation (via upregulating cell cycle genes), inhibits apoptosis (via modulating BCL-2/Caspase pathways), and enhances steroid synthesis (via regulating StAR, CYP11A1, and CYP19A1), ultimately supporting follicular selection, growth, and maturation.

Theoretically, this study expands the understanding of non-coding RNA-mediated regulation in avian reproductive biology. While circRNA/miRNA/mRNA networks have been well-documented in mammalian folliculogenesis, their roles in poultry—economically critical avian species—remain understudied. Our identification of the circFBN1/miR-206/E2F5 axis demonstrates that ceRNA networks are also key regulators of chicken GC function, suggesting evolutionary conservation of this regulatory mechanism across vertebrates. Additionally, our findings highlight the specificity of circRNA function: circFBN1, derived from exons 3–6 of the FBN1 gene (which encodes a structural glycoprotein involved in cell proliferation), exerts a regulatory role distinct from its linear parent gene—indicating that circRNAs are not just “byproducts” of transcription but functional molecules with unique roles in reproductive physiology. Practically, the circFBN1/miR-206/E2F5 axis provides potential molecular targets for improving poultry reproductive performance. For example, overexpressing circFBN1 in SYFs could enhance GC proliferation and steroid synthesis, increasing the number of follicles that successfully pass selection and reducing atresia—ultimately improving egg production rates. Conversely, inhibiting miR-206 (e.g., via antagomirs) could achieve similar effects by upregulating E2F5.

In conclusion, this study systematically interprets the functional role of the circFBN1/miR-206/E2F5 axis in chicken GC proliferation, apoptosis, and steroid synthesis. By linking circRNA-mediated miRNA sponging to a transcription factor that controls key GC functions, our results provide a new molecular framework for understanding avian follicular development and offer promising targets for enhancing poultry reproductive performance.

## 4. Materials and Methods

### 4.1. Chicken Follicle Harvesting

Taihang chickens were obtained from Hebei Tiankai Poultry Industry Technology Co., Ltd.’s conservation base (Handan, China). Individually housed and maintained under uniform dietary and environmental conditions, the hens reached 43 weeks of age before humane euthanasia. Ovaries were then removed and rinsed with PBS (Invitrogen, Carlsbad, CA, USA). Small yellow follicles (SYFs) and large yellow follicles (LYFs) were collected for circRNA sequencing, with SYFs specifically used for granulosa cell (GC) isolation. The study utilized eighteen birds, allocating six per group for follicle collection.

### 4.2. CircFBN1 Validation and qRT-PCR

Further investigation focused on circFBN1, a circular RNA derived from FBN1 exons 3, 4, 5 and 6. Total RNA isolated from ovaries and follicles at various developmental grades enabled cyclization verification. Using the HiScript III 1st Strand cDNA Synthesis Kit (+gDNA wiper) (Vazyme, Nanjing, China), cDNA was generated. Amplification across the circular junction employed specifically designed primers, and PCR amplicons were validated by electrophoresis and sequencing (Table 1). To quantify expression differences of circFBN1 and FBN1 via qRT-PCR, SYF RNA underwent RNase R digestion. qPCR was performed with ChamQTM Universal SYBR^®^ qPCR Master mix (Vazyme, Nanjing, China), using 18s rRNA and GAPDH for normalization. Melting curves ensured specific amplification. The 2^−ΔΔCT^ approach calculated relative quantities [46], with each analysis repeated three times.

### 4.3. Plasmid Construction and Dual-Luciferase Reporter Assay

The complete circFBN1 sequence was synthesized and inserted into the psicheck2 vector (Promega, Madison, WI, USA) to generate a reporter plasmid for dual-luciferase assays. Potential interacting miRNAs were predicted by matching the circRNA sequence against the seed region (nt 2–7/8), critical for miRNA-target binding. Bioinformatics analysis identified miR-206 as circFBN1 targets. To validate binding, specific mutations were engineered within the miR-206 binding sites to create mutant plasmids. Subsequently, 293T cells seeded in 96-well plates underwent transfection using GenXPIII Transfection Reagent (Probe Gene, Xuzhou, China). After 48 h, luciferase activity was quantified with the Dual-Luciferase^®^ Reporter Assay System (Promega, Madison, WI, USA). All assays included triplicate runs alongside control groups: empty plasmid (positive) and plasmid co-transfected with scrambled miRNA (negative).

### 4.4. GC Isolation and Culture

Granulosa cells (GCs) were harvested from small yellow follicles (SYFs) using a modified protocol based on Gilbert et al. [47]. Cell density and viability were determined via Trypan blue exclusion (Sigma-Aldrich, St. Louis, MO, USA). Isolated cells underwent culture in M199 medium (Gibco, Grand Island, NY, USA) supplemented with 10% fetal calf serum (FCS; Gibco) at 37 °C within a humidified CO_2_ incubator (Thermo Fisher Scientific, Waltham, MA, USA). Three independent biological replicates were analyzed for every experiment.

### 4.5. Cell Transfection

The CircFBN1 sequence was inserted into the pcDNA 3.1(+)-circ Mini Vector (Invitrogen, Carlsbad, CA, USA) using NheI/ApaI restriction sites. Subsequently, granulosa cells (GCs) underwent transfection employing Lipofectamine 3000 reagent (Invitrogen). This process involved introducing the empty vector (pcDNA 3.1(+)-circ Mini), the CircFBN1-expressing construct (pcDNA 3.1(+)-circ Mini circFBN1), specific miRNA mimics, and negative control mimics (mimics NC). All transfections were replicated three times independently.

### 4.6. Cell Proliferation Assay

Cell suspensions were washed in PBS and density-adjusted to 1 × 10^6^/mL. Centrifugation of 100 µL aliquots (250× *g*, 5 min) preceded supernatant removal; pellets were reconstituted in 20 µL binding buffer. Sequential staining involved: (1) 10 min incubation with 5 µL Annexin V-FITC (Invitrogen, Australia), followed by (2) addition of 10 µL propidium iodide (PI; Invitrogen) and 5 min dark incubation at RT.

### 4.7. Cell Apoptosis Assay

The Annexin V-FITC/PI Apoptosis Detection Kit (Vazyme, Nanjing, China) facilitated apoptotic measurement by flow cytometry. Cells underwent manufacturer-prescribed processing and staining procedures. FlowJo_V10 (BD Biosciences, Bedford, MA, USA) analyzed the acquired datasets. Each condition was tested in triplicate, incorporating positive (apoptosis-induced) and negative (untreated) controls.

### 4.8. Western Blot

Transfected primary chicken granulosa cells were harvested 48 h post-transfection and lysed using Beyotime buffer (Shanghai, China) supplemented with Roche protease inhibitors (Basel, Switzerland). Protein samples (30 µg) were denatured in loading buffer (50 mM Tris HCl pH 6.8, 2% SDS, 100 mM DTT, 10% glycerol, 0.1% bromphenol blue) via boiling, separated on 10% SDS-polyacrylamide gels by electrophoresis, and transferred to membranes. Immunoblotting employed specific primary antibodies (all Proteintech, Wuhan, China): anti-CDK2 (19532-1-AP; 1:1000), anti-PCNA (19532-1-AP; 1:1000), anti-caspase-3 (25128-1-AP;1:1000), anti-StAR (12225-1-AP; 1:1000), anti-CYP11A1(13363-1-AP;1:1000) and anti-β-tubulin (10094-1-AP; 1:2000). After primary incubation and washing, blots were treated with Proteintech secondary antibody (SA00001-2; 1:2000). Signal visualization utilized an ECL system (Thermo Fisher Scientific, Waltham, MA, USA). Densitometry analysis involved background subtraction, normalization of target protein intensity to β-tubulin, and expression of this ratio relative to untreated controls (defined as 100%). Results shown are representative of three independent replicate experiments.

### 4.9. ELISA Assay for Steroid Hormones

Following centrifugation at 3000 rpm for 20 min, the cell supernatant was collected from each well. Subsequently, the concentrations of steroid hormones, including estradiol (E2) and progesterone (P4), were measured in accordance with the protocols specified in the ELISA kits for chicken hormones, which were supplied by Jiangsu Meimian Industrial Co., Ltd., located in Yangcheng, China.

### 4.10. Statistical Analyses

Data are represented as mean ± standard error of the mean (SEM). Statistical significance was assessed using SPSS 20.0 software (IBM, Armonk, NY, USA) with a threshold of *p* < 0.05. Graphs were constructed using GraphPad Prism version 8.0.2 (GraphPad Software, San Diego, CA, USA). Each experiment included a minimum of three biological replicates.

## 5. Conclusions

**Figure 9:**
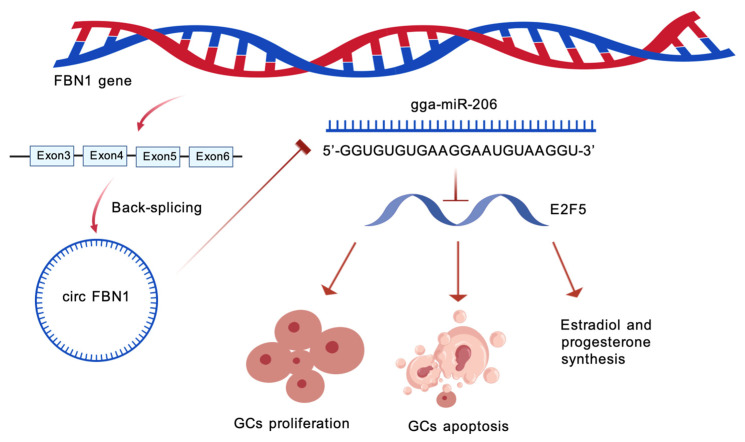
The mechanism by which circFBN1 regulates chicken granulosa cell proliferation, apoptosis, and E2/P4 synthesis through the miR-206/E2F5 signaling pathway.

In summary, this experiment constructed a complete regulatory pathway: circFBN1 adsorbs miR-206, preventing its binding to E2F5 and maintaining the high expression level of E2F5. E2F5 further influences the proliferation, apoptosis and steroid hormone synthesis processes of granulosa cells by regulating the expression of cell cycle-related genes, apoptosis-related genes and steroid synthesis-related genes, ultimately providing support for the selection, growth and maturation of chicken follicles. The results of this study not only reveal the significant roles of circFBN1, ga-mir-206 and E2F5 in chicken follicular granulosa cells, but also clarify their interaction mechanisms (Figure 9). These results offer a new perspective on understanding the molecular regulatory network of follicular development in poultry and provide potential molecular targets for the genetic improvement of poultry reproductive performance.

## Figures and Tables

**Table 1:** Characteristics of study subjects.

Primer	Sequence	Annealing Temp (°C)	Product Size (bp)
circFBN1	F: GAACGCATTGTGGACAGCCT R: GGCTATCTGACCATTTGGGC	60	274
CYP19A1	F: GGCCTCCAGCAGGTTGAAAG R: ATAGGCACTGTGGCAACTGG	60	214
E2F5	F: GCCTTCCAGACTCAGTGTTG R: GGCTCCTCCATCTTTGCTAT	60	148
CDK1	F: TGGCCTTGAACCACCCATAC R: AGGCAGGCAGGCAAAGATAA	60	298
CCND1	F: ATAGTCGCCACTTGGATGCT R: AACCGGCTTTTCTTGAGGGG	60	122
CYP11A1	F: GTGGACACGACTTCCATGACT R: GAGAGTCTCCTTGATGGCGG	60	174
PCNA	F: AACACTCAGAGCAGAAGAC R: GCACAGGAGATGACAACA	60	225
Caspase-9	F: GCTTGTCCATCCCAGTCCAA R: CAGTCTGTGGTCGCTCTTGT	60	95
Caspase-3	F: TGGCCCTCTTGAACTGAAAG R: TCCACTGTCTGCTTCAATACC	60	139
BCL2	F: ATCGTCGCCTTCTTCGAGTT R: ATCCCATCCTCCGTTGTTCT	60	150
StAR	F: AACCTGCTTCACTCTGTATC R: CTCATTAACTTCCTCTTGTCTC	60	151
β-actin	F: GTCCACCGCAAATGCTTCTAA R: TGCGCATTTATGGGTTTTGTT	60	78
FSHR	F: AACCTGCTTCACTCTGTATC R: CTCATTAACTTCCTCTTGTCTC	60	184
PGDFA	F: GATGAGCGCAACGTGAGAAC R: CACCACTGATCCGGACAACA	60	106
SBF2	F: GATGAGCGCAACGTGAGAAC R: CACCACTGATCCGGACAACA	60	154
ANDKKIA	F: AGAAGGTGGTTATGCTGTG R:TAAGAAGGAATGCGAGGAAT	60	235

## Data Availability

The original contributions presented in this study are included in the article. Further inquiries can be directed to the corresponding author.
